# Abortive transcripts in exosomes - A potential biomarker for Alzheimer’s disease

**DOI:** 10.1371/journal.pone.0357752

**Published:** 2026-09-11

**Authors:** Haizhu Wu, Na Zhang, Weiwei Yan, Cailin Li, Xiaomei Zhang, Xianglin Xv, Fang Fang, Shaowei Qin, Lifeng Zhao

**Affiliations:** 1 Guilin Tourism University, School of Food and Health, Guilin, China; 2 Guilin Tourism University, Guangxi Engineering Research Center for Large-Scale Preparation & Nutrients and Hygiene of Guangxi Cuisine, Guilin, China; 3 Guilin Tourism University, Food Science and Technology Research Center, Guilin, China; 4 Guilin Medical University, School of Basic Medical Sciences, Guilin, China; Nelson Mandela African Institute of Science and Technology, TANZANIA, UNITED REPUBLIC OF

## Abstract

Diagnosis of Alzheimer’s disease (AD) relies on invasive cerebrospinal fluid analysis or costly neuroimaging, underscoring the need for minimally invasive blood-based biomarkers for early detection. Exosomes, promising biomarker carriers, are unexplored for ultra-short RNA species like abortive transcripts (ATs). We investigated whether 8-nucleotide ATs are selectively packaged into exosomes to reflect brain transcriptional dysregulation in AD. Using a transgenic AD mouse model and Aβ-stimulated BV2 microglia coupled with Base-Stacking Hybridization Assisted Ligation (BSHAL), we detected significant dysregulation of ATs from AD-relevant genes (*Nefl*, *Bace1*, *Tyrobp*, *Ccl2*, *Pf4*) in brain tissue; specifically, *Bace1*, *Tyrobp*, and *Pf4* ATs showed robust increases (2.21-fold to 17.50-fold). These dysregulated AT signatures were mirrored in peripheral blood exosomes: *Bace1*, *Tyrobp*, and *Pf4* ATs increased 10.58-, 38.72-, and 11.63-fold, respectively. Aβ-activated microglia demonstrated exosome-specific enrichment of ATs, particularly *Pf4* ATs (~3510-fold increase), confirming active exosomal packaging. Exosomal ATs confer cell-type specificity for central nervous system pathology, lipid bilayer-enhanced stability, and sensitivity to transcriptional changes preceding pathological aggregation. Our findings, obtained in a transgenic mouse model and an in vitro microglial system, establish exosomal ATs as a novel class of blood-based biomarkers with potential for early AD diagnosis, pending validation in human cohorts.

## 1. Introduction

Alzheimer’s disease (AD) is a progressive neurodegenerative disorder whose biological definition is based on the National Institute on Aging-Alzheimer’s Association (NIA-AA) Research Framework, which establishes the core pathological features of AD as extracellular senile plaques formed by abnormal deposition of β-amyloid (Aβ) and intracellular neurofibrillary tangles caused by hyperphosphorylated tau protein [1]. Traditional AD diagnosis primarily relies on clinical symptoms and neuropsychological assessments, but these methods have significant limitations. Clinical diagnosis is often established only at advanced disease stages when irreversible neuronal damage has already occurred, resulting in high misdiagnosis rates and an inability to distinguish AD from other neurodegenerative diseases [2]. Neuropsychological tests (such as MoCA) demonstrate insufficient predictive specificity for the mild cognitive impairment (MCI) stage [3], proving that traditional methods cannot accurately identify the conversion from MCI to AD and are difficult to use for early AD diagnosis. Current clinical early diagnosis of AD mainly relies on imaging techniques combined with cerebrospinal fluid biomarker detection methods [4–6]. However, due to the high cost and radiation exposure associated with imaging detection, it is difficult to apply these methods to patients with mild cognitive impairment and for early AD diagnosis [5,6]. Although cerebrospinal fluid can truly reflect pathophysiological changes in the central nervous system, it requires invasive lumbar puncture procedures for collection, and repeated cerebrospinal fluid collection poses significant physical and psychological challenges for patients, making it unsuitable for widespread application. These deficiencies have prompted the emergence of novel blood biomarker research. Bhatnagar et al. discovered significantly altered microRNAs in the plasma of AD patients [7], while Jiang and Guo et al. found that plasma proteins can serve as biomarkers to predict dementia or AD, but these studies are still in early stages, and their application prospects remain to be observed [8,9].

In research on plasma biomarkers, exosomes have become a hotspot as important mediators of intercellular communication, with their biogenesis, cargo selection, release, and uptake mechanisms becoming key areas of biomarker research [10]. Rajendran et al. first demonstrated in 2006 that AD β-amyloid peptides are co-released with exosomes, a pioneering discovery that provided new perspectives for understanding Alzheimer’s disease pathological mechanisms [11]. Dinkins et al. further clarified the crucial role of exosomes in disease development through their summary of research on exosomes and AD pathogenesis [12]. Statistical data show that recent studies on AD and blood microRNAs have used various sample types, with whole blood accounting for 39.59%, serum for 27.09%, and serum exosomes comprising 4.17%, making them common sample types [13]. In studies of exosomes as diagnostic tools for AD, Abner et al. found that multiple neuron-derived exosomal proteins were significantly increased in the plasma of AD patients, offering possibilities for developing non-invasive diagnostic methods [14]. Agliardi et al. confirmed that neuron-derived exosomes carry SNAP-25 in serum and represent a potential biomarker for AD, highlighting the value of exosomes in AD diagnosis [15]. Pluta et al. proposed that exosomes serve both as potential transmission factors and as potential biomarkers for Alzheimer’s disease, emphasizing their dual role in disease progression [16]. However, current research still faces challenges: first, there is a lack of studies on blood-isolated brain-derived exosomes in AD patients; such research would help develop brain-derived exosomal contents that better capture the complex multifactorial pathological features of AD, thereby providing more sensitive blood biomarkers [17]. Second, protein and conventional RNA biomarkers suffer from issues such as limited variety, low sensitivity, poor reproducibility, and insufficient specificity [18]. Therefore, new biomarkers and analytical methods are needed to provide reliable tools for early diagnosis of AD [19]. A recent study by Wu et al. demonstrated that integrating plasma cfRNA-seq data with brain-derived scRNA-seq data can identify a panel of 34 genes that distinguishes AD patients from healthy controls, highlighting the potential of blood-based transcriptomic biomarkers for AD screening [20].

Although non-coding RNAs represented by miRNAs are current research hotspots in biomarker applications, a special class of non-coding RNAs—abortive transcripts (ATs)—has received little attention [13,18]. ATs refer to nascent RNAs of 2–10 nucleotides released by RNA polymerase before synthesizing functional RNAs[21, 22]. It has been established that abortive initiation occurs with every round of transcription in organisms that employ RNA polymerases, and ATs can accumulate to detectable levels *in vivo* [21]. Despite decades of research since their discovery, the biological significance and function of ATs remain unknown, although some investigators have speculated that they may play a role in gene regulation [18]. The naturally occurring AT has a length of 2–10 nt, with 2–8 nt being more abundant. AT sequences longer than 10 nt, even up to 19 nt, are only observed in some initiation sequences and genes with mutated discriminator sequences [22,23,24]. As byproducts of full-length RNA transcription, their occurrence and abundance are positively correlated with the transcription levels of homologous RNAs (RNAs capable of producing these ATs during transcription), with occurrence amounts being tens to hundreds of times greater than those of homologous RNAs. Therefore, changes in RNA transcription levels caused by pathological alterations *in vivo* will also lead to changes in the abundance of their homologous ATs[18, 21]. RNAseq has shown that 2770 genes undergo differential RNA expression in the brain tissue of AD patients, potentially causing changes in the transcription levels of more than 2000 ATs. Moreover, their small molecular weight makes ATs more easily enter the peripheral blood, making them potential AD diagnostic biomarkers [18,25]. However, because ATs are too short to be qualitatively and quantitatively detected *in vivo*, research on ATs as biomarkers remains limited [18]. We previously developed a method termed Base-Stacking Hybridization Assisted Ligation (BSHAL) that can detect 4–10 nt ATs with high sensitivity and specificity, and can also distinguish ATs from RNA degradation fragments in biological samples. Using this method, we demonstrated that 8-nt ATs in plasma can serve as biomarkers for hepatocellular carcinoma in mice [18].We chose 8 nt ATs for further investigation, as they exhibit markedly higher specificity than 4–7 nt ATs and possess a specificity level comparable to 9 nt and 10 nt ATs; meanwhile, 8 nt ATs present a far higher abundance than their 9 nt and 10 nt counterparts [18,24]. Notably, despite the relatively high specificity of 8 nt ATs — with specificity herein defined as exclusive derivation from a single gene — absolute sequence uniqueness cannot be fully ensured. Taking the human genome as an example, according to the research findings of the Encyclopedia of DNA Elements (ENCODE) Project, there are 62,403 transcription start sites (TSSs) in the human genome [26]. Theoretically, there are 65,536 possible types of 8 nt AT sequences. Therefore, if the first 8 nucleotides upstream of all transcription start sequences are non-repetitive, the number of distinct 8 nt AT sequences will exactly match the number of TSSs. However, in our previous study, we found that some AT sequences are specific, originating from a single gene, while others are shared by multiple genes. However, we also found that ATs without strict specificity were also differentially expressed in the plasma of hepatocellular carcinoma (HCC) mice. This may be attributed to the fact that other genes generating the same AT are lowly expressed or transcriptionally silent in the target tissues and biofluids [18]. For this study, we preferentially selected ATs with demonstrated gene specificity. Given that RNA exists mainly as low-molecular-weight fragments in human plasma with miRNAs being the most abundant, and that exosomes are important carriers for RNA transport in plasma, combined with the potential of ATs as biomarkers, detecting ATs in exosomes may become a novel approach for early diagnosis of AD, offering potential pathways for non-invasive detection of neurodegenerative diseases.

## 2. Materials and methods

### 2.1. Animals

Thirty-three-week-old male transgenic mice (Strain name: B6/JGpt-Tg(Thy-APP/Thy-PSEN1)5/Gpt; Strain abbreviation: FAD4T; Strain type: Tg; Strain number: T053302; Genetic background: C57BL/6JGpt) were utilized in this study. These mice, purchased from GemPharmatech Co., Ltd.(China), harbor both the human APP gene carrying Swedish and Indiana mutations and the human PSEN1 gene with M146V and L286V mutations. All experiments were performed per ARRIVE guidelines, related regulations, and the American Veterinary Medical Association (AVMA) Guidelines for Animal Euthanasia. The Institutional Animal Care and Use Committee of Guilin Tourism University reviewed and approved the animal experiment proto-col(Approval No. 2023280021). All mice (n = 30) were housed under strictly controlled environmental conditions maintained at 22 ± 2°C with 50% ± 5% relative humidity and a 12-hour light/dark cycle. Animals had ad libitum access to autoclaved water and standard chow. To minimize animal suffering, mice were monitored daily for signs of distress or discomfort, and cage enrichment materials were provided throughout the housing period. Spatial learning and memory capacities were longitudinally assessed throughout the feeding period. Following 33 weeks of intervention, mice were subjected to an 8-hour fasting period and then deeply anesthetized by intraperitoneal injection of sodium pentobarbital (50 mg/kg body weight). Adequate depth of anesthesia was confirmed by the absence of pedal withdrawal reflex prior to any invasive procedure. After blood collection, anesthetized mice were euthanized by cervical dislocation, a method consistent with AVMA guidelines for rodent euthanasia. Blood samples collected via orbital puncture were centrifuged (3,000 rpm, 10 min, 4°C) to isolate serum, which was subsequently stored at −80°C. Brains were rapidly excised on ice and sagittally bisected along the midline. The left hemispheres were flash-frozen in liquid nitrogen for biochemical analyses, while the right hemispheres were immersion-fixed in histological preservative (Solarbio, China) prior to paraffin embedding and sectioning.

### 2.2. Evaluation of spatial learning and memory ability in mice

The Morris water maze test comprehensively evaluated mice’s spatial learning and memory ability, including positioning, navigation, and space exploration. To ensure the reliability of the experimental results, the experiment was carried out at a fixed time every day to reduce the experimental error caused by the biological clock and other factors. One day before the experiment, the mice were taken to the operating room and allowed to move freely in the operating room for a period of time to familiarize themselves with the operating environment. Then, the mice were put into the water to swim freely, familiarize them with the water environment, and reduce the influence of the stress response caused by the unfamiliar environment on the experimental results.

### 2.3. Morphological examination of hippocampal neurons in mice

The brain tissues of the mice were taken for paraffin-embedded sections, stained with HE, and observed under an inverted microscope, and the morphology of neurons in the hippocampus of the mice was recorded.

### 2.4 Preparation of Aβ_1–42_ oligomers

According to Wan’s experimental method [27], 1 mg Aβ_1–42_ protein (GL Biochem, China) was dissolved in 22 μL of precooled hexafluoroisopropanol (HFIP) (Macklin, China). The mixture was vortexed and incubated at room temperature until fully dissolved in an ice bath. The solution was dissolved in DMSO (Macklin, China) to a concentration of 5 mmol/L, and the concentration was adjusted to 100μmol/L with precooled F-12 medium (Procell, China). After vortexing and mixing, the mixture was incubated at 4°C for 24h, centrifuged at 13000g for 10 min, and the supernatant was stored at −80 °C until use.

### 2.5. Cell Culture and in vitro model construction

BV2 cells were resuscitated with F12 medium. After 5–10 passages, they were cultured in exosome-free fetal bovine serum (Procell, China) F12 medium and co-cultured with 100μM Aβ_1–42_ oligomers for 24h. Cells in the control group were cultured synchronously using F12 medium.

### 2.6. Model exosomes from peripheral blood and BV2 cells were isolated

After mice were anesthetized, peripheral blood samples were collected in EDTA anticoagulant tubes and centrifuged at 3000 rpm, 5 min at 4°C. The supernatant was transferred to a new centrifuge tube, and exosomes were extracted with Total Exosome Isolation (Invitrogen, USA). The upper culture medium was aspirated 24h after the in vitro cell model was established, and exosomes were extracted from the cell culture supernatant using Total Exosome Isolation (Invitrogen, USA).

### 2.7. The morphology of exosomes was identified

A sample of 10 μL of exosomes was dropped onto the copper mesh for precipitation for 1 min, and then 10 μL of uranyl acetate (EMS, USA) was dropped onto the copper mesh for precipitation for 1 min. The floating liquid was sucked off with filter paper and dried at room temperature for several minutes.

### 2.8. The particle size of exosomes was detected

After thawing in a water bath at 25°C, frozen samples of exosomes were diluted in 1 × PBS (Sangon Biotech, China) and used for the NTA assay (PMX120, Germany). The results were analyzed using ZetaView.

### 2.9. Western Blot

The exosomes were mixed with the lysate containing PMSF (Keygen Biotech, China) and Cocktail (Sigma, Germany), lysed for 30 min on ice, and centrifuged at 14000 rpm for 5 min at 4°C. The centrifuged supernatant was divided into aliquots and transferred to a clean centrifuge tube. The mixture was mixed with 5 × loading buffer at 4:1, boiled for 10 min, slowly restored to room temperature, slightly centrifuged, and stored at −20°C. Protein concentration was quantified using a Bicinchoninic acid (BCA) kit (Keygen Biotech, China). Proteins were separated by SDS-PAGE and transferred to Immobilon-P Transfer Membrane (PVDF) (MILLIPORE, Germany). The membranes were rinsed in TBST for 5 min, then infiltrated in 5% skim milk powder solution, and blocked for 2h at room temperature. At the end of blocking, the cells were rinsed with TBST for 8 min and then incubated with primary antibodies overnight at 4°C. The membrane was incubated with the secondary antibody for 1h at 37°C and then washed 3 times with TBST. The hybrid membrane was placed on a transparent plastic plate and reacted with the chemiluminescence substrate for 3 min. The development was completed in a darkbox, and the band results on the bottom plate film were scanned. The information on the antibodies used in the WB experiment steps can be found in Table S1 in S1 File of the supplementary file.

### 2.10. The 8nt ATs sequence of the differential gene was determined

Studies have shown that *Nefl* [28], *Bace1* [29], *Tyrobp* [30], *Ccl2* [31], *Pf4* [32], *Acp2* [33], *Bcar3* [34], and *Bnip3* [35] are specifically expressed in Alzheimer’s disease. Use the DBTSS database TSS-Viewer tool (https://kero.hgc.jp/tool/tss_search.html) to retrieve each gene transcription start position and determine the length of the 8nt ATs base sequence. The initial transcription sequences of each gene, which are 8nt in length, can be found in supplementary file Table S2.

These eight genes were selected based on two criteria. First, each gene has been independently reported to be dysregulated in Alzheimer’s disease brain tissue or AD-relevant cell types, with the supporting references cited above. Second, an experimentally validated transcription start site was available in the DBTSS database for each gene, which was necessary for the unambiguous design of the splint DNA probes used in the BSHAL assay. Genes that met the first criterion but lacked a well-supported TSS annotation were excluded, because probe design under TSS uncertainty would compromise detection specificity. The eight genes span diverse functional categories relevant to AD pathogenesis, including Aβ processing, microglial signaling, neuroinflammation, and axonal integrity, and represent a targeted panel intended for proof-of-principle demonstration rather than an unbiased screening effort.

### 2.11. Real-time fluorescence quantitative PCR was used to detect the differential gene expression in the brain tissue of AD model mice

Total RNA was extracted from mouse brain tissue using the RNAsimple Total RNA Kit (Tiangen, China). The One-Step gDNA Removal and cDNA Synthesis SuperMix (Transgene, China) kit was used to reverse transcribe total RNA into cDNA using Oligo dT as a primer, which was used as a template. Real-time PCR was performed using PerfectStart® Green qPCR SuperMix (Transgene, China) and CFX Connect (Bio-Rad, USA). *Gapdh* was used as the reference gene to calculate the relative mRNA expression changes relative to the control group. The sequences of the primers used in each gene qPCR reaction are presented in Table S3 of the supplementary file.

### 2.12. Small RNA was extracted from brain tissue and exosomes

Long fragments of RNA were separated from the filtrate containing small RNA using a CR3 adsorption column in the RNAsimple Total RNA Kit (Tiangen, China), and small RNA was precipitated using 2.5 times absolute ethanol with 1/10 sodium acetate.

### 2.13. The abortive transcript content was detected by TaqMan-MGB qPCR

The BSHAL technique achieves length-specific detection of 8-nucleotide ATs through the design of the splint DNA (SPD). Each SPD consists of three contiguous segments: 20 nucleotides complementary to the upstream adapter RNA (ARU), a central region of exactly eight nucleotides complementary to the target AT, and 20 nucleotides complementary to the downstream adapter RNA (ARD). Hybridization of ARU and ARD to their respective SPD segments creates an 8-nucleotide gap that precisely accommodates the target AT. T4 DNA ligase catalyzes ligation only when the 5′-monophosphate of ARD and the 3′-hydroxyl of ARU are positioned immediately adjacent to the termini of the correctly hybridized AT, with no intervening gap or overhang. ATs that are shorter or longer than eight nucleotides fail to meet this geometric requirement and cannot be efficiently ligated. As previously validated [18], ATs differing by a single nucleotide in length produce no detectable signal when assayed against an SPD designed for an 8nt target.

In addition to length specificity, the BSHAL assay distinguishes authentic ATs from RNA degradation fragments by exploiting a difference in their 5′ phosphorylation states. ATs are synthesized de novo by RNA polymerases and carry a 5′-triphosphate group, whereas degradation products generated by cellular nucleases bear a 5′-monophosphate. To eliminate degradation fragments, extracted small RNA is first treated with Calf Intestinal Alkaline Phosphatase (CIP, NEB, USA), which removes 5′-monophosphates but does not act on 5′-triphosphates. After heat inactivation of CIP, polyphosphatase is used to convert the 5′-triphosphate of authentic ATs to a 5′-monophosphate, the substrate required by T4 DNA ligase in the subsequent ligation reaction. This two-step enzymatic treatment ensures that only genuine ATs, and not degradation fragments, are captured and detected.

The sequence of splint DNAs (SPD) was designed according to the Base-Stacking Hybridization Assisted Ligation (BSHAL) method established in Qin [18] to capture the corresponding ATs, and the MGB probe designed for the starting transcription sequence of each gene was used to detect the 8nt ATs content level of each gene by qPCR. The adapter RNAs sequence and SPD sequence are respectively presented in Table S4 and Table S5 of the supplementary file. The fluorescence probe sequence and primer sequence of TaqMan-MGB qPCR are respectively listed in Table S6 and Table S7 of the supplementary file.

## 3. Results

### 3.1. Validation of Alzheimer’s disease transgenic mouse model

**Fig 1:**
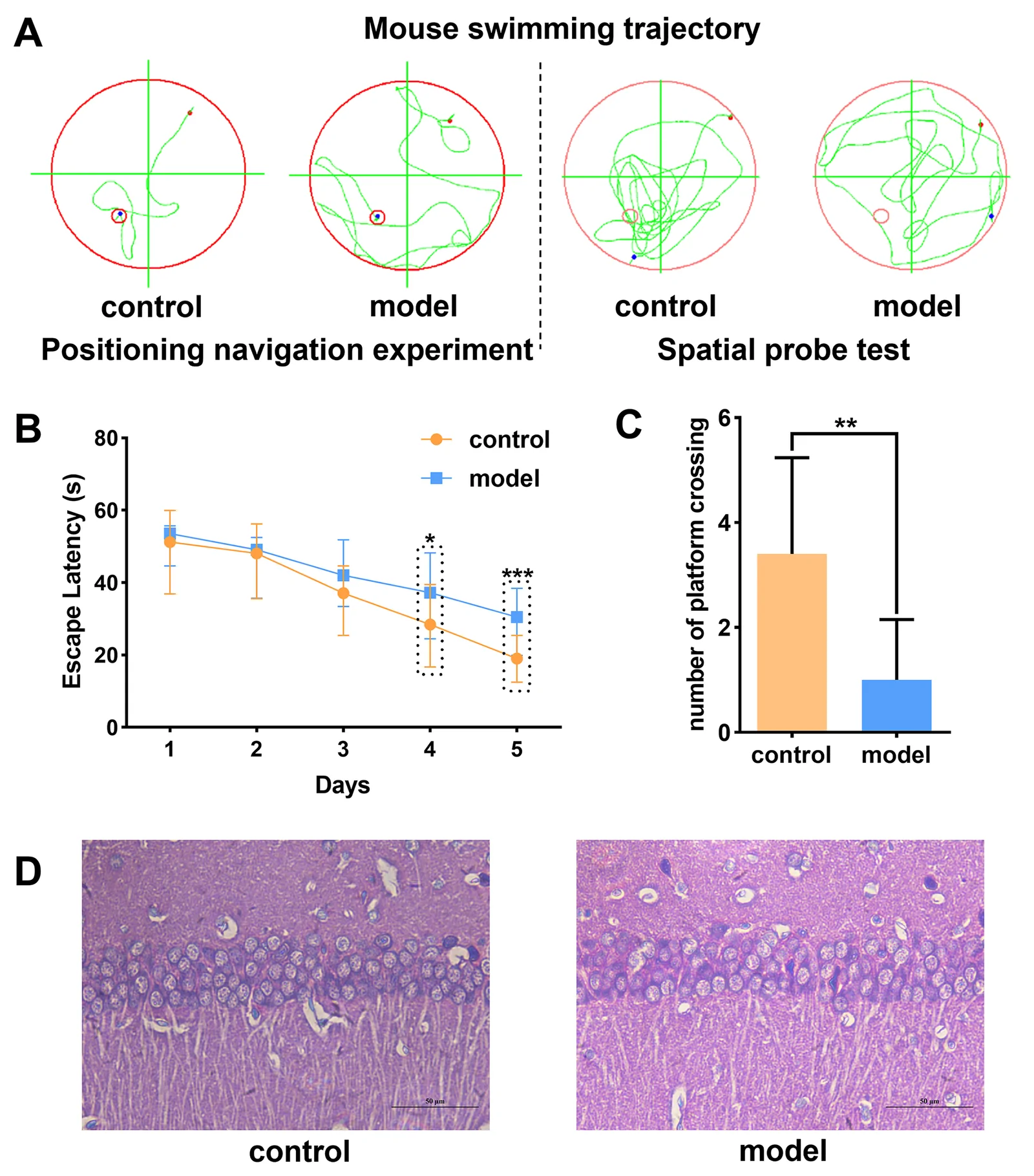
Evaluation and identification of AD mouse models. (A) Swimming trajectory of mice in the Morris water maze test. (B) The average escape latency of the model and control groups in the localization navigation experiment. *p *<* 0.05, ***p *<* 0.001. (C) The number of platform crossings between the model and control groups in the space exploration experiment. ***p *<*. (D) HE staining results of hippocampal neurons in each group (high power lens 400×).

Morris water maze analysis confirmed that transgenic AD mice exhibit significantly impaired learning and memory relative to wild-type controls. During place navigation training, escape latency progressively shortened in both groups across training days. However, AD models demonstrated sustained latency impairment, with maximal divergence by day 5 (model 29.43 ± 6.89 vs control 18.00 ± 6.68 days, Fig 1B). Swim trajectory analysis revealed predominant thigmotactic navigation (wall-hugging) in transgenic mice versus goal-directed paths in controls (Fig 1A). Probe trials showed severely reduced platform crossings in AD models (1.30 ± 1.50) versus controls (3.80 ± 1.54, Fig 1C). Histological assessment of hippocampal CA1 neurons demonstrated preserved cytoarchitecture in controls (spherical/pyriform cells with defined margins), whereas transgenic specimens exhibited nuclear pyknosis, cytoplasmic vacuolation, and neuronal loss with structural disintegration (Fig 1D). These findings establish that transgenic-induced CA1 dysfunction mediates spatial memory deficits, faithfully recapitulating AD histopathological and behavioral phenotypes for mechanistic investigation.

### 3.2. Peripheral blood exosomes transport brain-derived ATs as alzheimer’s biomarkers

**Fig 2:**
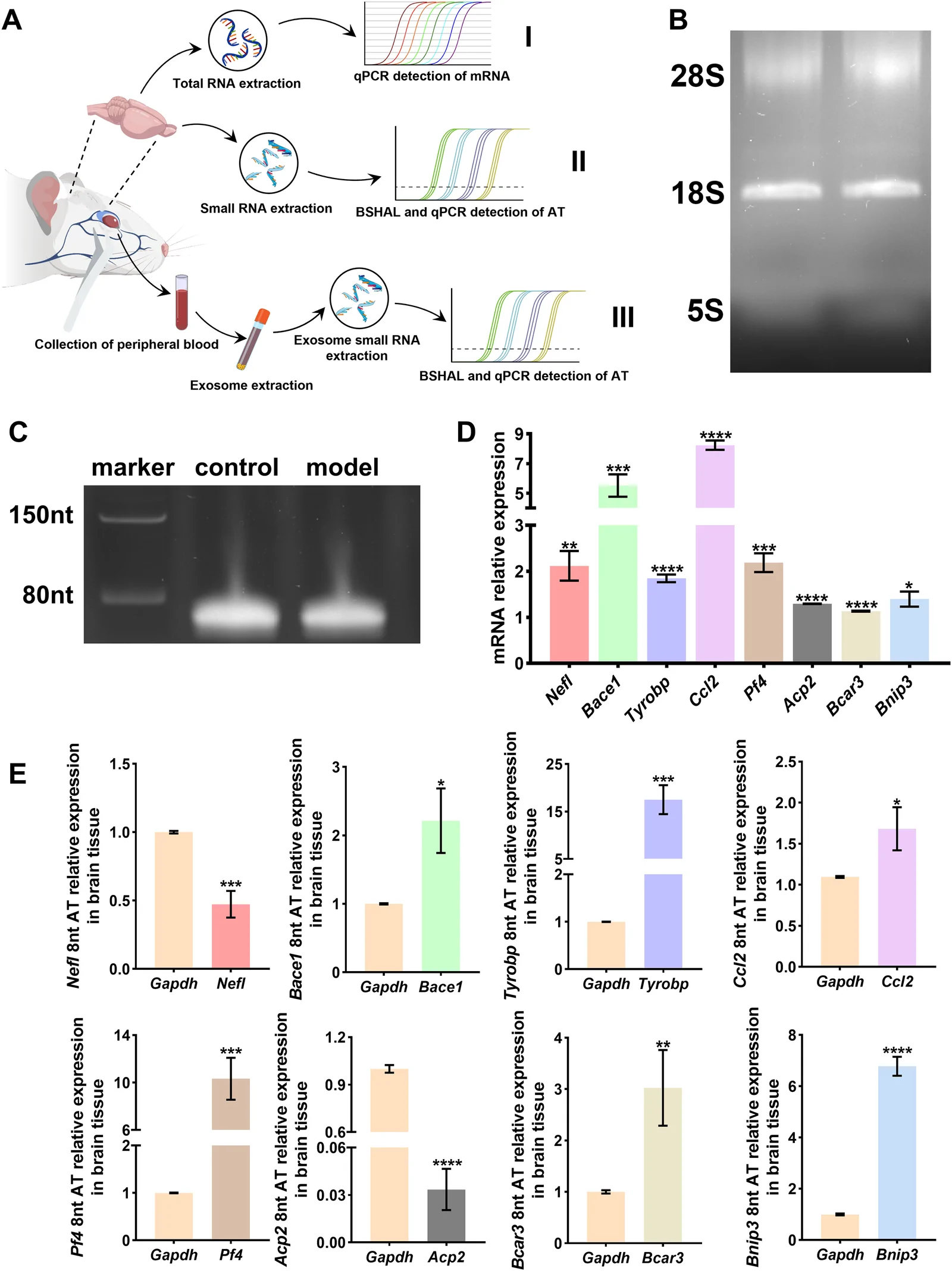
Detect the AD-related differential genes and the content of 8nt ATs in the brain tissues of AD model mice. (A) Schematic representation of mRNA and small RNA detection in mouse brain tissue and peripheral blood. (B) 1% agarose gel electrophoresis of total mouse RNA. (C) Detection of small RNA in mouse brain tissue by Urea polyacrylamide gel electrophoresis. (D) RT-qPCR results of each differentially expressed gene mRNA in mouse brain tissue *p *<* 0.05, **p *<* 0.01, ***p *<* 0.001, ****p *<* 0.0001. (E) Quantification of 8-nucleotide ATs in brain tissue of control and AD model mice. Relative AT abundance was determined by BSHAL coupled with TaqMan-MGB qPCR. All values were normalized to the 8-nt AT of *Gapdh* as an internal reference and are expressed as fold change relative to the control group. *p *<* 0.05, **p *<* 0.01, ***p *<* 0.001, ****p *<* 0.0001.

To investigate whether ATs could be stably detected in peripheral blood following potential vesicular transport from brain tissues, we first analyzed total RNA and small RNA fractions (< 80nt) isolated from the brains of control and AD model mice (Fig 2B, C). Quantitative assessment of disease-relevant transcripts revealed significant upregulation (*p* < 0.01 for all) of mRNA levels in AD brain tissues versus controls. Specifically, expression increased 2.12-fold for *Nefl*, 5.52-fold for *Bace1*, 1.85-fold for *Tyrobp*, 8.24-fold for *Ccl2*, 2.19-fold for *Pf4*, 1.30-fold for *Acp2*, 1.13-fold for *Bcar3*, and 1.4-fold for *Bnip3* (Fig 2D). BSHAL assays were performed using gene-specific SPDs with an 8-nucleotide central complementary region, ensuring length-specific detection as described in the Methods. Subsequent quantification of 8nt ATs using BSHAL assays identified divergent abundance patterns: while *Nefl* and *Acp2* ATs decreased significantly (to 0.474-fold and 0.0335-fold, respectively), *Bace1*, *Tyrobp*, *Ccl2*, *Pf4*, *Bcar3*, and *Bnip3* ATs were markedly elevated (2.21-fold, 17.50-fold, 1.68-fold, 10.33-fold, 3.023-fold, and 6.78-fold, respectively) (Fig 2E). These data confirm transcriptional dysregulation and selective accumulation of ATs species in AD-affected brain parenchyma.

**Fig 3:**
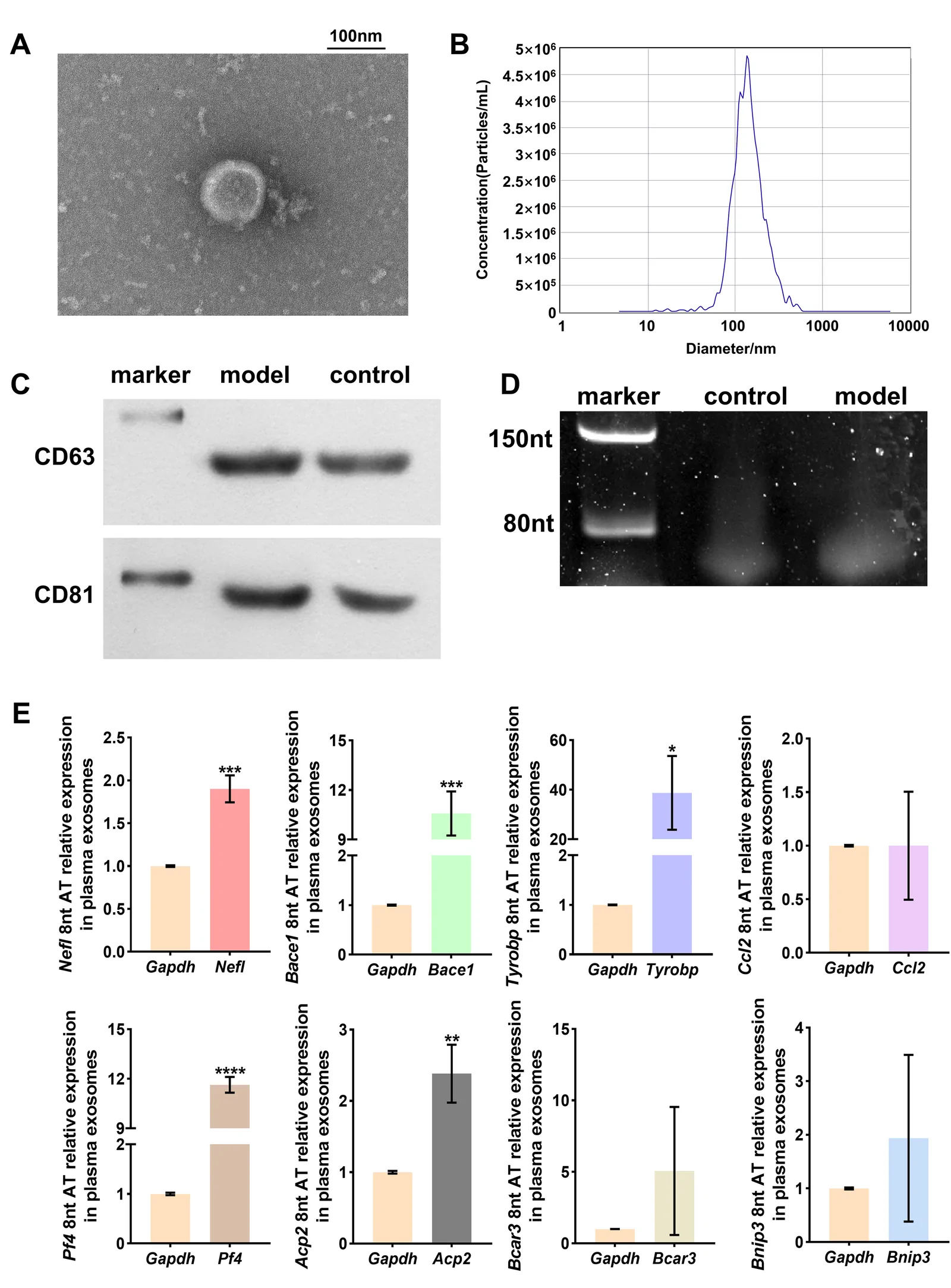
Detect the changes in the 8-nucleotide ATs content of multiple genes in the exosomes of peripheral blood from AD model mice. (A) The morphology of exosomes in peripheral blood photographed by TEM. (B) NTA was used to detect the particle size distribution and concentration of exosomes in peripheral blood. (C) Western Blot identification of exosome-associated proteins CD63 and CD81. (D) Small RNA of exosomes from the peripheral blood of mice was detected by Urea polyacrylamide gel electrophoresis. (E) Quantification of 8-nucleotide ATs in peripheral blood exosomes of control and AD model mice. Relative AT abundance was determined by BSHAL coupled with TaqMan-MGB qPCR. All values were normalized to the 8-nt AT of *Gapdh* as an internal reference and are expressed as fold change relative to the control group. *p *<* 0.05, **p *<* 0.01, *** *p* *<* 0.001, ****p *<* 0.0001.

To assess the potential exosome-mediated translocation of ATs to systemic circulation, we isolated extracellular vesicles from murine peripheral blood. Transmission electron microscopy (TEM) imaging revealed characteristic spherical vesicles with an approximate diameter of 100 nm (Fig 3A). Nanoparticle tracking analysis (ZetaView) further quantified vesicle parameters, demonstrating a mean particle size of 155.9 nm and a concentration of 1.1 × 10^11^ Particles/mL (Fig 3B). Western blot analysis confirmed robust enrichment of canonical exosomal markers CD63 and CD81 (Fig 3C), validating successful vesicle isolation. We subsequently extracted small RNAs from blood-derived exosomes and quantified gene-specific 8nt ATs using the BSHAL assay. Analysis revealed statistically significant elevations of specific ATs species in AD model mice relative to controls: *Nefl* ATs abundance increased 1.90-fold, *Bace1* increased 10.58-fold, *Tyrobp* increased 38.72-fold, *Pf4* increased 11.63-fold, and *Acp2* increased 2.38-fold (Fig 3E). In contrast, ATs levels of *Ccl2*, *Bcar3*, and *Bnip3* exhibited no significant alterations. Critically, directional changes in ATs abundance for *Bace1*, *Tyrobp*, *Pf4*, and *Acp2* in blood exosomes exhibited consistency with dysregulation patterns observed in brain tissues—specifically, concurrent increases in both compartments for *Bace1*, *Tyrobp*, and *Pf4*, while *Acp2* ATs decreased in brain but increased in circulation (Fig 2E vs. 3E). Collectively, these results provide empirical support for exosome-facilitated transport of ATs from neural tissues to peripheral blood in AD pathogenesis. The notable concordance between tissue-specific AT alterations and exosomal profiles validates the potential utility of bloodborne exosomal ATs as clinically accessible biomarkers for AD.

### 3.3. Exosome-Mediated ATs Transfer from Aβ_1-42_-Activated Microglia Generates Blood AD Biomarkers

**Fig 4:**
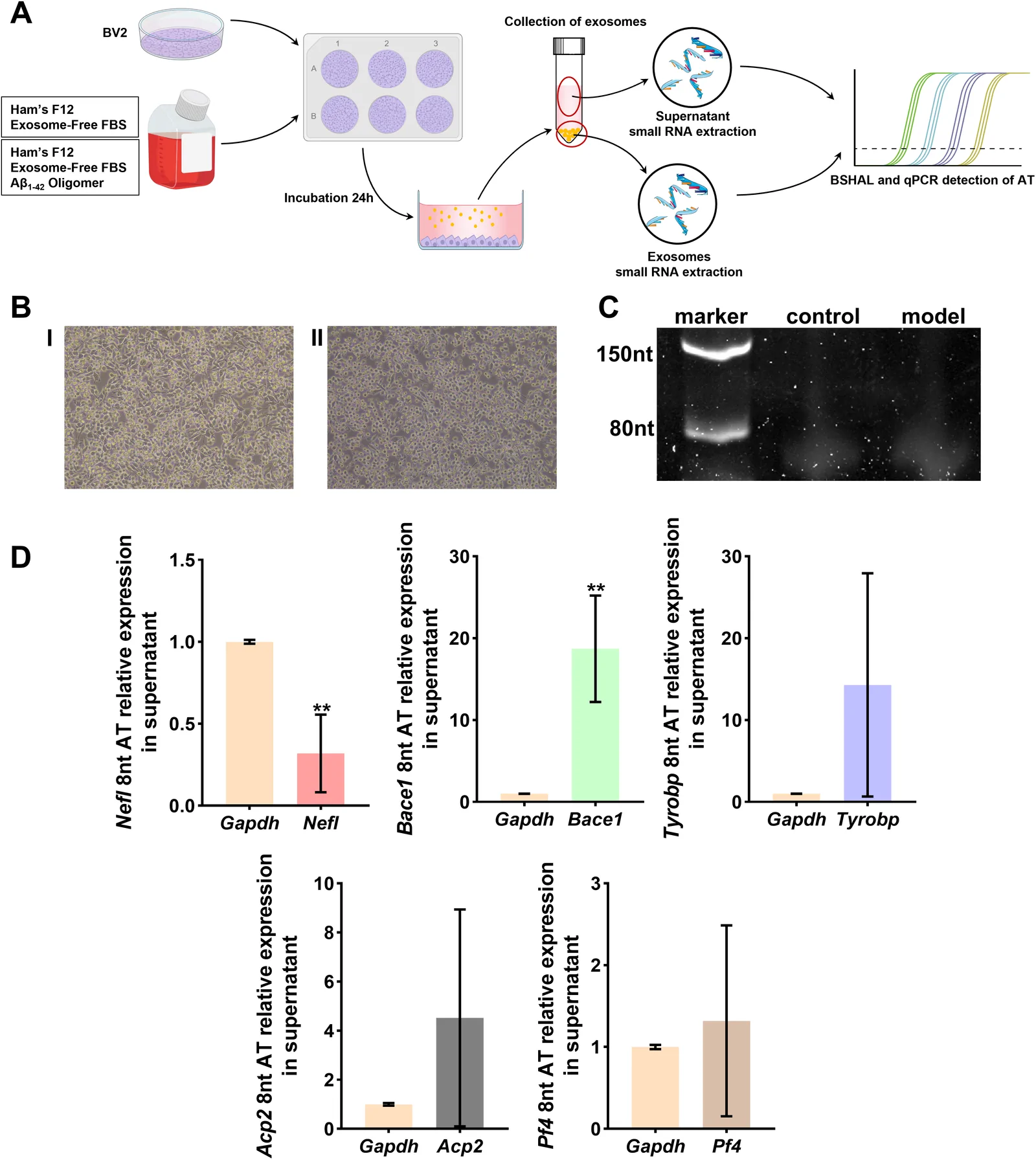
Detect the content of 8-nucleotide ATs of AD-related genes in the supernatant of the BV2 cell in vitro model after the removal of exosomes. (A) Schematic representation of ATs detection in each AD in vitro cell model part. (B) I. Untreated BV2 cells; II. Aβ_1-42_ oligomer induced BV2 cells. (C)Small RNA in the exosomes-free supernatant medium of BV2 cells was detected by urea polyacrylamide gel electrophoresis. (D) Quantification of 8-nucleotide ATs in exosome-depleted supernatant of Aβ ~ 1-42 ~ -stimulated BV2 microglia. Relative AT abundance was determined by BSHAL coupled with TaqMan-MGB qPCR. All values were normalized to the 8-nt AT of *Gapdh* as an internal reference and are expressed as fold change relative to the unstimulated control group. **p *<* 0.01.

**Fig 5:**
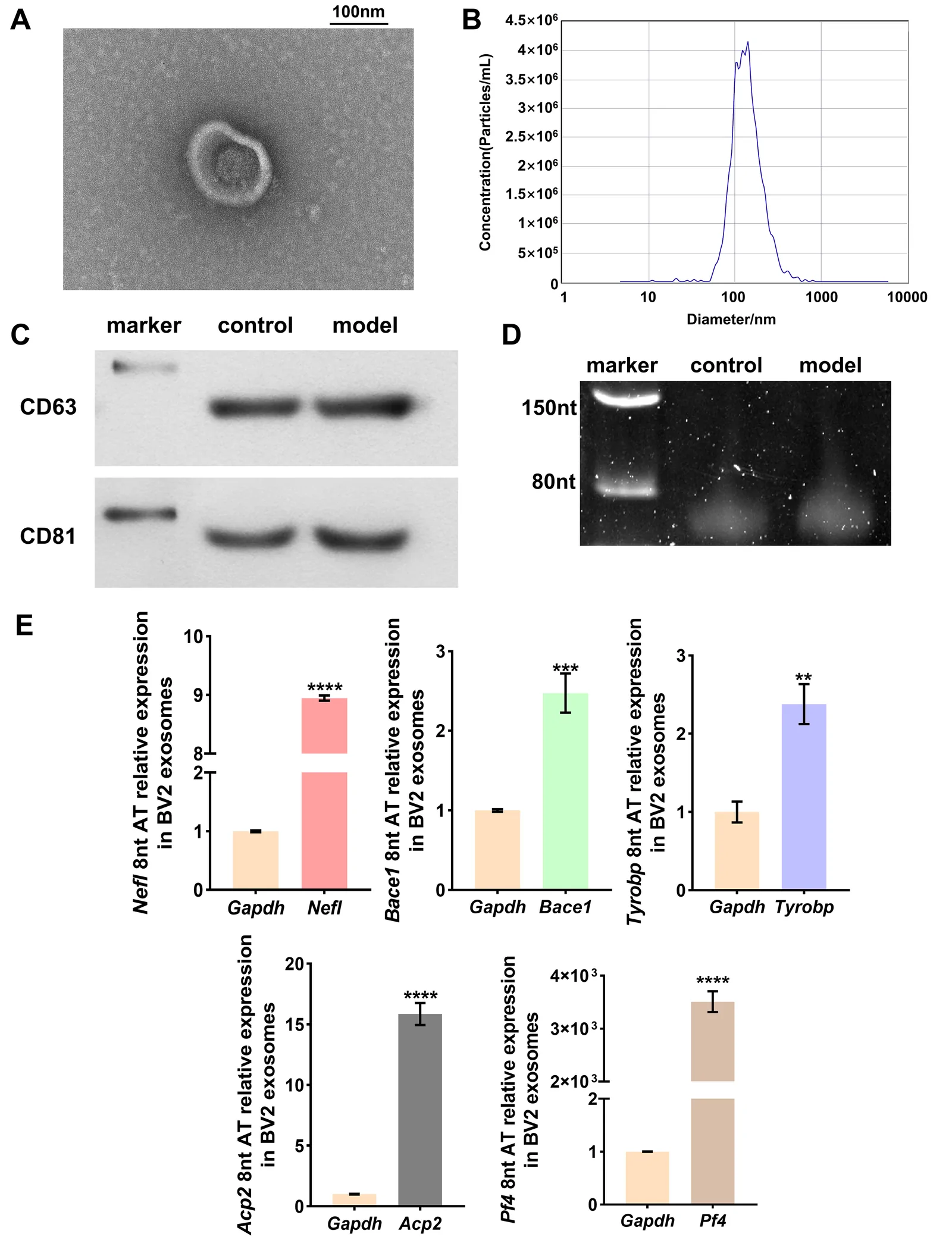
Detect the content of 8-nucleotide ATs in the exosomes of the supernatant of the BV2 cell in vitro model related to AD genes. (A) The morphology of exosomes in the supernatant of BV2 cells was photographed by TEM. (B) NTA was used to detect the particle size distribution and concentration of exosomes in the supernatant of BV2 cells. (C) Western Blot (WB) identification of exosome-associated proteins CD63 and CD81. (D) Small RNA of exosomes from the supernatant of BV2 cells was detected by Urea polyacrylamide gel electrophoresis. (E) Quantification of 8-nucleotide ATs in exosomes isolated from the supernatant of Aβ ~ 1-42 ~ -stimulated BV2 microglia. Relative AT abundance was determined by BSHAL coupled with TaqMan-MGB qPCR. All values were normalized to the 8-nt AT of *Gapdh* as an internal reference and are expressed as fold change relative to the unstimulated control group. **p *<* 0.01, ***p *<*, ****p *<* 0.0001.

To validate whether ATs are actively exported from cells via exosomes, we treated BV2 microglia with Aβ_1–42_ oligomers to establish an in vitro Alzheimer’s disease model. Following exosome isolation from conditioned supernatants, small RNAs were extracted separately from exosome-depleted supernatants and purified exosomes for ATs quantification (Fig 4A). Aβ_1–42_-exposed BV2 cells exhibited reduced adhesion, increased suspension, and morphological shifts toward rounded phenotypes with fewer polygonal/spindle-shaped cells, consistent with microglial activation. In exosome-free supernatants extracted for small RNA (Fig 4C), quantitative BSHAL assays detected significant AT alterations for only *Nefl* and *Bace1*: *Nefl* decreased 0.319-fold, while *Bace1* increased 18.72-fold (Fig 4D), revealing partial and directionally inconsistent changes compared to in vivo blood exosomal AT signatures. Transmission electron microscopy of BV2-derived exosomes confirmed characteristic spherical vesicles <200 nm in diameter (Fig 5A). In comparison, nanoparticle tracking analysis (ZetaView) quantified a mean size of 150 nm and concentration of 1.0 × 10^11^ Particles/mL (Fig 5B). Western blotting verified robust expression of exosomal markers CD63 and CD81 (Fig 5C), confirming successful vesicle isolation. Critically, BSHAL analysis of exosomal small RNAs (Fig 5D) demonstrated significant ATs enrichment in Aβ_1–42_-treated cells versus controls: *Nefl* increased 8.95-fold (*p* < 0.0001), *Bace1* increased 2.38-fold, *Tyrobp* increased 2.38-fold, *Pf4* increased 3.51 × 10^3^-*fold*, and *Acp2* increased 15.85-fold (Fig 5E). This coordinated upregulation confirms efficient exosomal export of stable ATs. Critically, the directional patterns—specifically, *Bace1* elevation, *Tyrobp* elevation, *Pf4* elevation, and *Acp2* elevation—exhibited complete congruence with ATs changes detected in peripheral blood exosomes from AD mice. This concordance mechanistically supports exosome-mediated ATs translocation from neural cells into systemic circulation, reinforcing their biomarker utility for Alzheimer’s disease diagnostics.

## 4. Discussion

Recent intensive research on extracellular vesicles in neurodegenerative diseases has revealed their dual role as dissemination factors and biomarkers [36,37], providing essential context for investigating ATs within exosomes; while methodological advances through BSHAL technology, which enables qualitative and quantitative detection of ATs, have established the methodological foundation for this study [18]. This work provides the first direct evidence that ATs - 8nt RNA fragments produced during transcription initiation – are selectively packaged into peripheral blood exosomes as molecular mirror images of central nervous system transcriptional dysregulation during Alzheimer’s disease pathogenesis. Using transgenic AD models and Aβ-stimulated BV2 microglial cells, we demonstrated significant consistency in key AD-associated AT signatures (particularly *Bace1*, *Tyrobp*, and *Pf4*) between brain tissue and blood-derived exosomes (Fig 3E, 5E). This finding directly addresses critical limitations in current AD diagnostics: plasma p-tau217 biomarkers remain unreliable for preclinical detection due to variability, while established methods like CSF Aβ/tau testing and amyloid-PET involve invasive procedures or high costs [38–40]. Exosomal ATs overcome these barriers through three inherent advantages: cell-type specificity reflecting neuronal/glial stress within the CNS, exceptional stability within lipid bilayer vesicles [37,41], and sensitivity to transcriptional dysregulation – an event preceding insoluble protein aggregation [18,21]. This work addresses a crucial gap in neurodegenerative disease biomarker research: conventional extracellular vesicle (EV) RNA analyses have overlooked ultra-short RNA fragments (< 15nt), despite their potential to reveal molecular pathology preceding protein aggregation.

The extraordinary 3.51 × 10^3^ fold upregulation of *Pf4* ATs in BV2-derived exosomes is a remarkable finding and may reflect transcriptional dysregulation and efficient exosome packaging mechanisms in activated microglia. This unprecedented increase may be related to neuronal hyperexcitability in Alzheimer’s disease models or inflammatory responses that trigger specific transcriptional programs [40,42–44]. The extreme magnitude suggests a possible feedforward amplification mechanism or unusually efficient exosome sorting, possibly involving sequence-specific RNA-binding proteins that recognize the *Pf4* transcription initiation sequence. This magnitude of enrichment is unlikely to be explained solely by the 2.19-fold increase in *Pf4* mRNA we observed in brain tissue. *Pf4* encodes a chemokine associated with neuroinflammatory signaling and has been identified as a hub gene in AD transcriptional networks. During microglial activation, increased transcription initiation at the *Pf4* promoter, combined with the repetitive nature of abortive cycling, could generate a disproportionately large pool of *Pf4* ATs. Concurrent activation of exosome biogenesis pathways could facilitate the efficient export of these accumulated ATs. An alternative possibility is that the initial transcribed sequence of *Pf4* possesses structural features recognized by RNA-binding proteins involved in exosomal cargo selection, although the magnitude of enrichment substantially exceeds that typically reported for sequence-based sorting. Distinguishing between these possibilities will require further biochemical studies. This finding is consistent with the principle that any alterations in RNA transcription caused by pathological conditions should correspondingly affect AT production [18].

The divergent patterns of *Nefl* and *Acp2* 8-nt ATs—significantly downregulated in brain tissue (0.474-fold and 0.0335-fold, respectively) yet markedly upregulated in peripheral blood exosomes (1.90-fold and 2.38-fold, respectively)—warrant careful consideration. While it is possible that the ATs detected in this study represent random degradation fragments of longer RNAs, several lines of evidence argue against this interpretation. The BSHAL-based protocol used here includes an enzymatic pre-treatment step that removes the 5′-monophosphate from degradation fragments, preventing their ligation to the adapters and subsequent detection by qPCR. Furthermore, if the detected ATs were merely degradation products, one would expect comparable levels between the control and experimental groups across all genes, which is not what we observed. Alternatively, blood–brain barrier leakage has been documented in patients with early Alzheimer’s disease. We speculate that such leakage may allow a greater number of exosomes carrying brain-derived ATs to enter the peripheral circulation, thereby elevating exosomal AT levels for certain genes even when their tissue levels are reduced. This could explain why *Nefl* and *Acp2* ATs, despite being downregulated in the brain tissue of AD mice, appear at higher levels in peripheral blood exosomes due to increased passage of brain-derived exosomes into the circulation [45]. We acknowledge that this interpretation is based on prior findings and theoretical considerations, and the underlying mechanisms await further experimental validation.

However, limitations of this study must be acknowledged. One is to consider that the heterogeneity of exosome subsets and the complex mechanisms governing RNA packaging pose challenges for clinical translation [41,46]. Future research should focus on standardising exosome isolation protocols, utilizing microfluidic platforms for exosome detection to facilitate translation to clinical applications [37,47,48] and enable precise characterization of extracellular vesicles in neurodegenerative diseases [46,49,50]. The second is to consider differences in human cohorts. Recent studies demonstrating the demographic and clinical characteristics of AD patients and normal controls highlight the importance of well-characterized patient cohorts for biomarker validation [37,51]. The differences in mean age and clinical characteristics between the AD and control groups highlight the need for age-matched controls and careful consideration of confounding factors in future studies.

It should also be noted that the present findings were obtained from a transgenic mouse model and an immortalized BV2 microglial cell line. The FAD4T model recapitulates key features of AD including amyloid pathology and neuroinflammation, but does not capture the full complexity of sporadic human AD, which involves advanced age, vascular comorbidities, and substantial inter-individual variability. Future studies in well-characterized human cohorts, incorporating age-matched controls and standardized exosome isolation protocols, will be needed to determine whether the exosomal AT signatures identified here can be translated into clinically useful biomarkers.

## 5. Conclusion

In summary, our study demonstrates that exosomal ATs represent a novel class of biomarkers with significant potential for the diagnosis and monitoring of Alzheimer’s disease. The selective packaging of specific AT species into exosomes provides critical insights into both disease mechanisms and potential diagnostic applications. The capability of the BSHAL technique, combined with TaqMan-MGB qPCR, to detect ATs with nucleotide-resolution sensitivity provides a robust platform for future biomarker research [18]. Further investigations are required to elucidate the biological functions of these ATs and to establish standardized protocols for clinical detection, particularly in light of the rapid advancements in extracellular vesicle research and their implications for neurodegenerative disease diagnostics. The integration of these novel biomarkers with existing diagnostic modalities, complemented by advanced computational methods and artificial intelligence, could ultimately lead to the development of a comprehensive diagnostic panel capable of accurately detecting Alzheimer’s disease at its earliest stages, thereby enabling timely intervention and improved patient prognosis.

## Supporting information

S1 File All relevant supporting data are provided in the supplementary files, which include information on the animals used in this research and Supplementary Tables. (ZIP)

S2 File Raw images. (PDF)

S1 Fig Graphical abstract. (TIF)

## References

[1] Jack CR Jr, Bennett DA, Blennow K, Carrillo MC, Dunn B, Haeberlein SB, et al. NIA-AA Research Framework: Toward a biological definition of Alzheimer’s disease. Alzheimers Dement. 2018;14(4):535–62. doi: 10.1016/j.jalz.2018.02.018 29653606 PMC5958625

[2] Jack CR Jr, Holtzman DM, Sperling R. Dementia is not synonymous with Alzheimer’s disease. Sci Transl Med. 2019;11(522):eaav0511. doi: 10.1126/scitranslmed.aav0511 31826979

[3] Chehrehnegar N, Nejati V, Shati M, Rashedi V, Lotfi M, Adelirad F, et al. Early detection of cognitive disturbances in mild cognitive impairment: a systematic review of observational studies. Psychogeriatrics. 2020;20(2):212–28. doi: 10.1111/psyg.12484 31808989

[4] Song J, Qin W, Liu Y, Duan Y, Liu J, He X, et al. Aberrant functional organization within and between resting-state networks in AD. PLoS One. 2013;8(5):e63727. doi: 10.1371/journal.pone.0063727 23667665 PMC3647055

[5] Bateman RJ, Xiong C, Benzinger TLS, Fagan AM, Goate A, Fox NC, et al. Clinical and biomarker changes in dominantly inherited Alzheimer’s disease. N Engl J Med. 2012;367(9):795–804. doi: 10.1056/NEJMoa1202753 22784036 PMC3474597

[6] Sperling R, Johnson K. Biomarkers of Alzheimer disease: current and future applications to diagnostic criteria. Continuum (Minneap Minn). 2013;19(2 Dementia):325–38. doi: 10.1212/01.CON.0000429181.60095.99 23558480 PMC10563888

[7] Bhatnagar S, Chertkow H, Schipper HM, Yuan Z, Shetty V, Jenkins S, et al. Increased microRNA-34c abundance in Alzheimer’s disease circulating blood plasma. Front Mol Neurosci. 2014;7:2. doi: 10.3389/fnmol.2014.00002 24550773 PMC3912349

[8] Jiang Y, Uhm H, Ip FC, Ouyang L, Lo RMN, Cheng EYL, et al. A blood-based multi-pathway biomarker assay for early detection and staging of Alzheimer’s disease across ethnic groups. Alzheimers Dement. 2024;20(3):2000–15. doi: 10.1002/alz.13676 38183344 PMC10984431

[9] Guo Y, You J, Zhang Y, Liu W-S, Huang Y-Y, Zhang Y-R, et al. Plasma proteomic profiles predict future dementia in healthy adults. Nat Aging. 2024;4(2):247–60. doi: 10.1038/s43587-023-00565-0 38347190

[10] Manna I, De Benedittis S, Quattrone A, Maisano D, Iaccino E, Quattrone A. Exosomal miRNAs as Potential Diagnostic Biomarkers in Alzheimer’s Disease. Pharmaceuticals (Basel). 2020;13(9):243. doi: 10.3390/ph13090243 32932746 PMC7559720

[11] Rajendran L, Honsho M, Zahn TR, Keller P, Geiger KD, Verkade P, et al. Alzheimer’s disease beta-amyloid peptides are released in association with exosomes. Proc Natl Acad Sci U S A. 2006;103(30):11172–7. doi: 10.1073/pnas.0603838103 16837572 PMC1544060

[12] Dinkins MB, Wang G, Bieberich E. Sphingolipid-Enriched Extracellular Vesicles and Alzheimer’s Disease: A Decade of Research. J Alzheimer’s Disease. 2017;60(3):757–68. doi: 10.3233/JAD-160567 27662306 PMC5360538

[13] Fattahi F, Asadi MR, Abed S, Kouchakali G, Kazemi M, Mansoori Derakhshan S, et al. Blood-based microRNAs as the potential biomarkers for Alzheimer’s disease: evidence from a systematic review. Metab Brain Dis. 2024;40(1):44. doi: 10.1007/s11011-024-01431-7 39607566

[14] Abner EL, Jicha GA, Shaw LM, Trojanowski JQ, Goetzl EJ. Plasma neuronal exosomal levels of Alzheimer’s disease biomarkers in normal aging. Ann Clin Transl Neurol. 2016;3(5):399–403. doi: 10.1002/acn3.309 27231710 PMC4863753

[15] Agliardi C, Guerini FR, Zanzottera M, Bianchi A, Nemni R, Clerici M. SNAP-25 in Serum Is Carried by Exosomes of Neuronal Origin and Is a Potential Biomarker of Alzheimer’s Disease. Mol Neurobiol. 2019;56(8):5792–8. doi: 10.1007/s12035-019-1501-x 30680692

[16] Pluta R, Ułamek-Kozioł M, Januszewski S, Czuczwar SJ. Exosomes as Possible Spread Factor and Potential Biomarkers in Alzheimer’S Disease: Current Concepts. Biomark Med. 2018;12(9):1025–33. doi: 10.2217/bmm-2018-0034

[17] Badhwar A, Haqqani AS. Biomarker potential of brain-secreted extracellular vesicles in blood in Alzheimer’s disease. Alzheimers Dement (Amst). 2020;12(1):e12001. doi: 10.1002/dad2.12001 32211497 PMC7085285

[18] Qin S, Wu H, Li C, Yang J, Yan W, He Z, et al. Detection of Naturally occurring abortive transcripts by Base-Stacking Hybridization Assisted Ligation and PCR amplification. Biosens Bioelectron. 2024;251:116099. doi: 10.1016/j.bios.2024.116099 38330773

[19] Li P, Kaslan M, Lee SH, Yao J, Gao Z. Progress in Exosome Isolation Techniques. Theranostics. 2017;7(3):789–804. doi: 10.7150/thno.18133 28255367 PMC5327650

[20] Wu L, Zhang R, Wang Y, Dai S, Yang N. Integrative single-cell and cell-free plasma RNA transcriptomics identifies biomarkers for early non-invasive AD screening. Front Aging Neurosci. 2025;17:1571783. doi: 10.3389/fnagi.2025.1571783 40520536 PMC12162594

[21] Goldman SR, Ebright RH, Nickels BE. Direct detection of abortive RNA transcripts in vivo. Science. 2009;324(5929):927–8. doi: 10.1126/science.1169237 19443781 PMC2718712

[22] Hsu LM. Monitoring abortive initiation. Methods. 2009;47(1):25–36. doi: 10.1016/j.ymeth.2008.10.010 18948204 PMC2647590

[23] Chander M, Lee A, Vallery TK, Thandar M, Jiang Y, Hsu LM. Mechanisms of Very Long Abortive Transcript Release during Promoter Escape. Biochemistry. 2015;54(50):7393–408. doi: 10.1021/acs.biochem.5b00712 26610896

[24] Brodolin K, Morichaud Z. Region 4 of the RNA polymerase σ subunit counteracts pausing during initial transcription. J Biol Chem. 2021;296:100253. doi: 10.1074/jbc.RA120.016299 33380428 PMC7948647

[25] Gns HS, Rajalekshmi SG, Burri RR. Revelation of Pivotal Genes Pertinent to Alzheimer’s Pathogenesis: A Methodical Evaluation of 32 GEO Datasets. J Mol Neurosci. 2022;72(2):303–22. doi: 10.1007/s12031-021-01919-2 34668150 PMC8526053

[26] ENCODE Project Consortium. An integrated encyclopedia of DNA elements in the human genome. Nature. 2012;489(7414):57–74. doi: 10.1038/nature11247 22955616 PMC3439153

[27] Wan W, Cao L, Liu L, Kalionis B, Chen C, Tai X, et al. EGb761 provides a protective effect against Aβ1-42 oligomer-induced cell damage and blood-brain barrier disruption in an in vitro bEnd.3 endothelial model. PLoS One. 2014;9(11):e113126. doi: 10.1371/journal.pone.0113126 25426944 PMC4245095

[28] Zhang T, Tang Y, Yang X, Wang X, Ding S, Huang K, et al. Expression of GSK3β, PICK1, NEFL, C4, NKCC1 and Synaptophysin in peripheral blood mononuclear cells of the first-episode schizophrenia patients. Asian J Psychiatr. 2021;55:102520. doi: 10.1016/j.ajp.2020.102520 33373836

[29] Beyer N, Coulson DTR, Quinn JG, Brockbank S, Hellemans J, Irvine GB, et al. mRNA levels of BACE1 and its interacting proteins, RTN3 and PPIL2, correlate in human post mortem brain tissue. Neuroscience. 2014;274:44–52. doi: 10.1016/j.neuroscience.2014.05.020 24853053

[30] Liu M, Li S, Ma C, Wang X, Shuang Y, Lin H, et al. Bioinformatics analysis indicates that microRNA‑628‑5p overexpression may alleviate Alzheimer’s disease by targeting TYROBP. Mol Med Rep. 2021;23(2):142. doi: 10.3892/mmr.2020.11781 33313948

[31] Pan H, Wang G, Bi Z, Lai C, Wang M. Identification of key neutrophil extracellular trap genes in Alzheimer’s disease. J Alzheimers Dis. 2024;102(4):1027–41. doi: 10.1177/13872877241295374 39584744

[32] Wang X, Wang L. Screening and Identification of Potential Peripheral Blood Biomarkers for Alzheimer’s Disease Based on Bioinformatics Analysis. Med Sci Monit. 2020;26:e924263. doi: 10.12659/MSM.924263 32812532 PMC7453750

[33] Xu M, Liu Q, Bi R, Li Y, Li H, Kang WB, et al. Coexistence of multiple functional variants and genes underlies genetic risk locus 11p11.2 of Alzheimer’s disease. Biol Psychiatry. 2023;94(9):743–59. doi: 10.1016/j.biopsych.2023.05.020 37290560

[34] Lv L, Zhang D, Hua P, Yang S. The glial-specific hypermethylated 3’ untranslated region of histone deacetylase 1 may modulates several signal pathways in Alzheimer’s disease. Life Sci. 2021;265:118760. doi: 10.1016/j.lfs.2020.118760 33212149

[35] Zhang S, Zhang Z, Sandhu G, Ma X, Yang X, Geiger JD, et al. Evidence of oxidative stress-induced BNIP3 expression in amyloid beta neurotoxicity. Brain Res. 2007;1138:221–30. doi: 10.1016/j.brainres.2006.12.086 17274962

[36] Arif S, Qazi TJ, Quan Z, Ni J, Li Z, Qiu Y, et al. Extracellular vesicle-packed microRNAs profiling in Alzheimer’s disease: The molecular intermediary between pathology and diagnosis. Ageing Res Rev. 2025;104:102614. doi: 10.1016/j.arr.2024.102614 39626853

[37] Kumar A, Su Y, Sharma M, Singh S, Kim S, Peavey JJ, et al. MicroRNA expression in extracellular vesicles as a novel blood-based biomarker for Alzheimer’s disease. Alzheimers Dement. 2023;19(11):4952–66. doi: 10.1002/alz.13055 37071449 PMC11663460

[38] Sethi P, Bhaskar R, Singh KK, Gupta S, Han SS, Avinash D, et al. Exploring advancements in early detection of Alzheimer’s disease with molecular assays and animal models. Ageing Res Rev. 2024;100:102411. doi: 10.1016/j.arr.2024.102411 38986845

[39] Qian Z, Li Y, Ye K. Advancements and challenges in mouse models of Alzheimer’s disease. Trends Mol Med. 2024;30(12):1152–64. doi: 10.1016/j.molmed.2024.10.010 39547883

[40] Li J, Liu Y, Yin C, Zeng Y, Mei Y. Structural and functional remodeling of neural networks in β-amyloid driven hippocampal hyperactivity. Ageing Res Rev. 2024;101:102468. doi: 10.1016/j.arr.2024.102468 39218080

[41] Cheng L, Vella LJ, Barnham KJ, McLean C, Masters CL, Hill AF. Small RNA fingerprinting of Alzheimer’s disease frontal cortex extracellular vesicles and their comparison with peripheral extracellular vesicles. J Extracell Vesicles. 2020;9(1):1766822. doi: 10.1080/20013078.2020.1766822 32922692 PMC7448944

[42] Ciccone R, Franco C, Piccialli I, Boscia F, Casamassa A, de Rosa V, et al. Amyloid β-Induced Upregulation of Nav1.6 Underlies Neuronal Hyperactivity in Tg2576 Alzheimer’s Disease Mouse Model. Sci Rep. 2019;9(1):13592. doi: 10.1038/s41598-019-50018-1 31537873 PMC6753212

[43] Busche MA, Eichhoff G, Adelsberger H, Abramowski D, Wiederhold K-H, Haass C, et al. Clusters of hyperactive neurons near amyloid plaques in a mouse model of Alzheimer’s disease. Science. 2008;321(5896):1686–9. doi: 10.1126/science.1162844 18802001

[44] Minkeviciene R, Rheims S, Dobszay MB, Zilberter M, Hartikainen J, Fülöp L, et al. Amyloid beta-induced neuronal hyperexcitability triggers progressive epilepsy. J Neurosci. 2009;29(11):3453–62. doi: 10.1523/JNEUROSCI.5215-08.2009 19295151 PMC6665248

[45] van de Haar HJ, Burgmans S, Jansen JFA, van Osch MJP, van Buchem MA, Muller M, et al. Blood-Brain Barrier Leakage in Patients with Early Alzheimer Disease. Radiology. 2016;281(2):527–35. doi: 10.1148/radiol.2016152244 27243267

[46] Li C, Wang W, Sun Y, Ni Y, Qin F, Li X, et al. Selective sorting and secretion of hY4 RNA fragments into extracellular vesicles mediated by methylated YBX1 to promote lung cancer progression. J Exp Clin Cancer Res. 2022;41(1):136. doi: 10.1186/s13046-022-02346-w 35410432 PMC8996536

[47] Tong Z, Xu X, Shen C, Yang D, Li Y, Li Q, et al. All-in-one multiple extracellular vesicle miRNA detection on a miniaturized digital microfluidic workstation. Biosens Bioelectron. 2025;270:116976. doi: 10.1016/j.bios.2024.116976 39591923

[48] Kim Y, Kim JY, Moon S, Lee H, Lee S, Kim JY, et al. Tumor-derived EV miRNA signatures surpass total EV miRNA in supplementing mammography for precision breast cancer diagnosis. Theranostics. 2024;14(17):6587–604. doi: 10.7150/thno.99245 39479442 PMC11519808

[49] Song S, Lee JU, Jeon MJ, Kim S, Sim SJ. Detection of multiplex exosomal miRNAs for clinically accurate diagnosis of Alzheimer’s disease using label-free plasmonic biosensor based on DNA-Assembled advanced plasmonic architecture. Biosens Bioelectron. 2022;199:113864. doi: 10.1016/j.bios.2021.113864 34890883

[50] Zhang J, Zhang Y, Wang J, Xia Y, Zhang J, Chen L. Recent advances in Alzheimer’s disease: Mechanisms, clinical trials and new drug development strategies. Signal Transduct Target Ther. 2024;9(1):211. doi: 10.1038/s41392-024-01911-3 39174535 PMC11344989

[51] Reho P, Kalia V, Jackson GL, Wang F, Eiten E, Brennan K, et al. Preclinical Alzheimer’s disease shows alterations in circulating neuronal-derived extracellular vesicle microRNAs in a multiethnic cohort. Alzheimers Dement. 2025;21(3):e70050. doi: 10.1002/alz.70050 40042514 PMC11881609

