# Supplementary material for: Abortive transcripts in exosomes - A potential biomarker for Alzheimer’s disease
Source: PLoS One. 2026 Sep 11;21(9):e0357752. doi: 10.1371/journal.pone.0357752 (PMC13567793; doi:10.1371/journal.pone.0357752)
Supplement: Supplementary file 1 [file pone.0357752.s002.pdf]

Original Images for Figure2.B

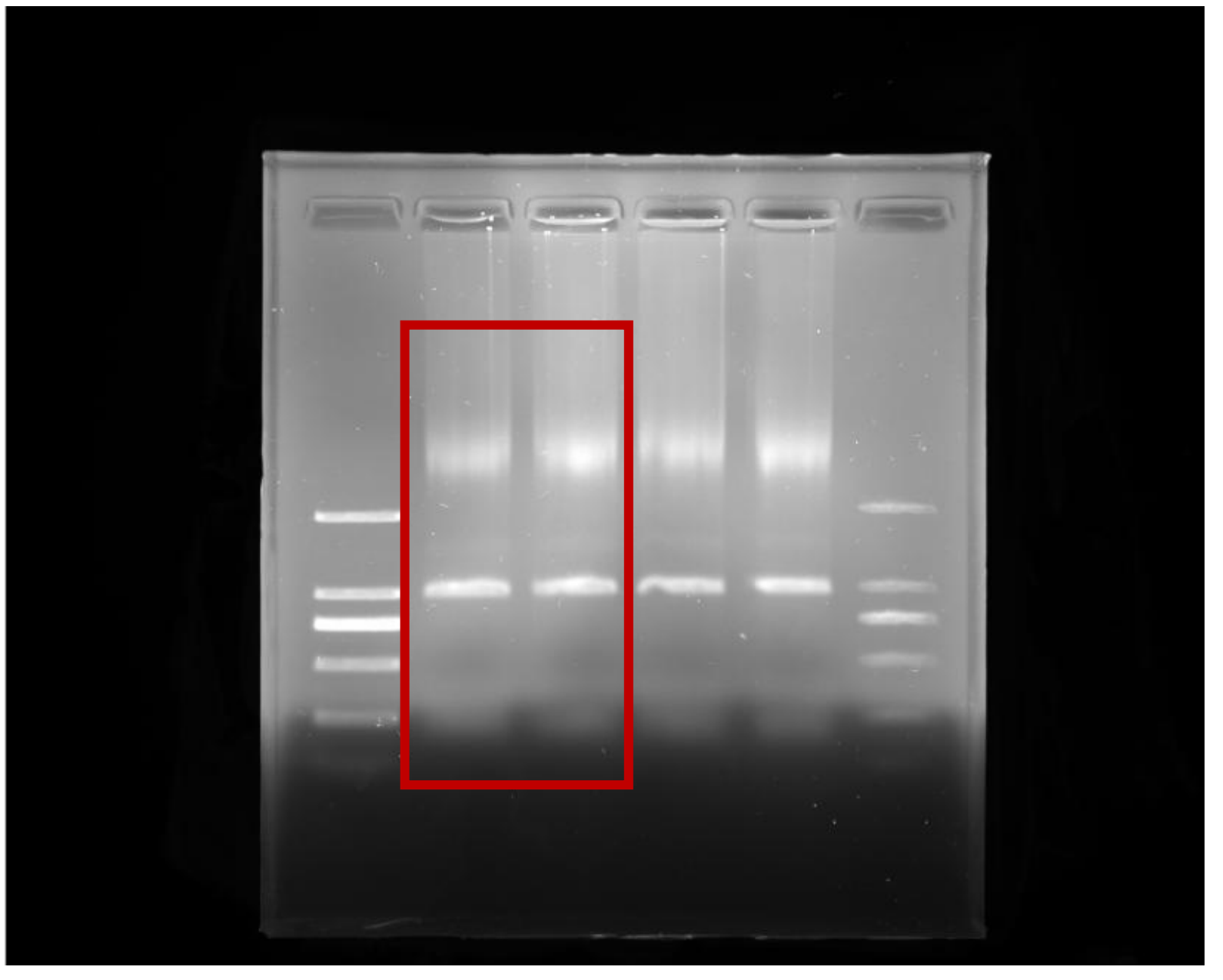

Original Images for Figure2.C

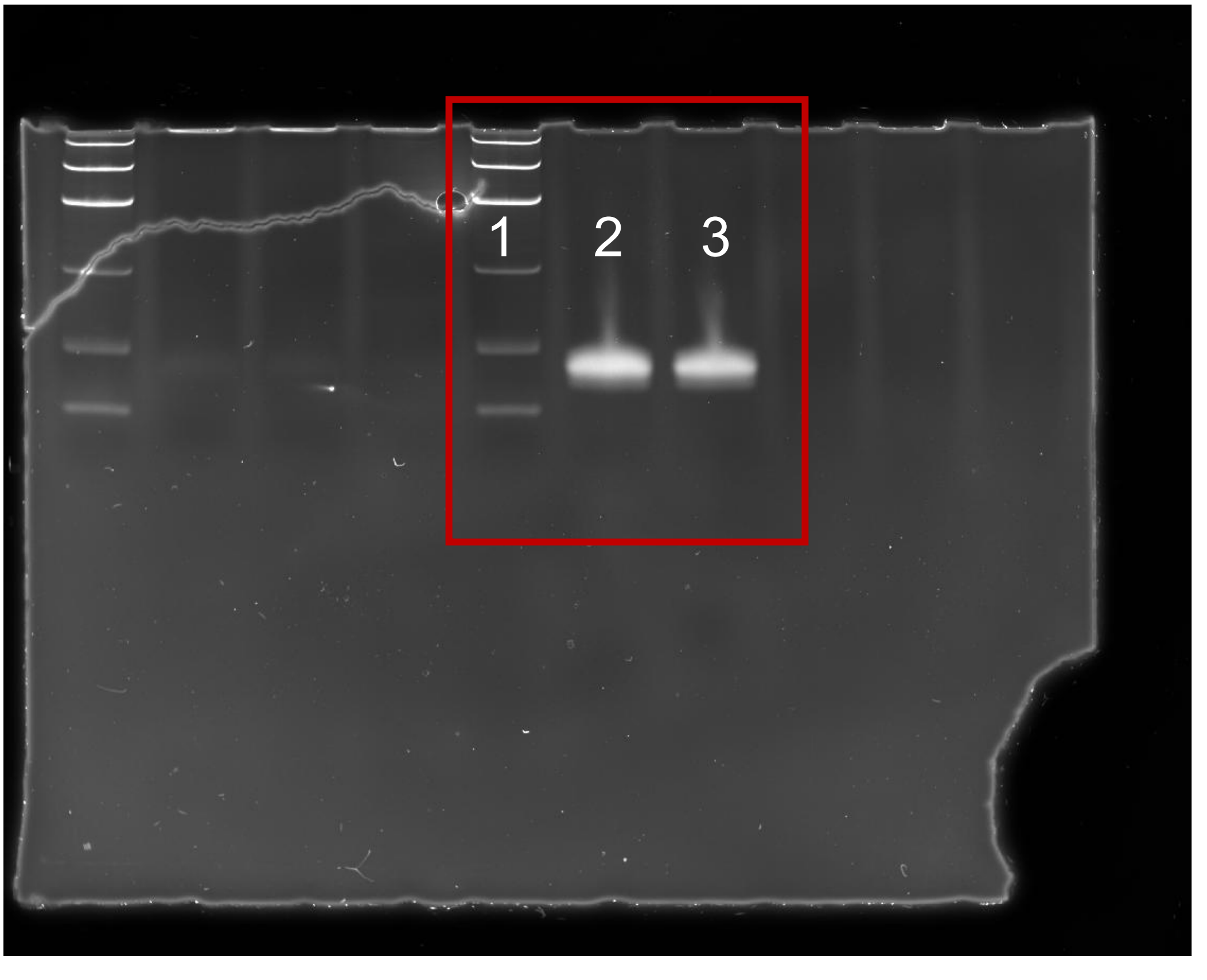

1. marker; 2. control; 3. model

Original Images for Figure3.A

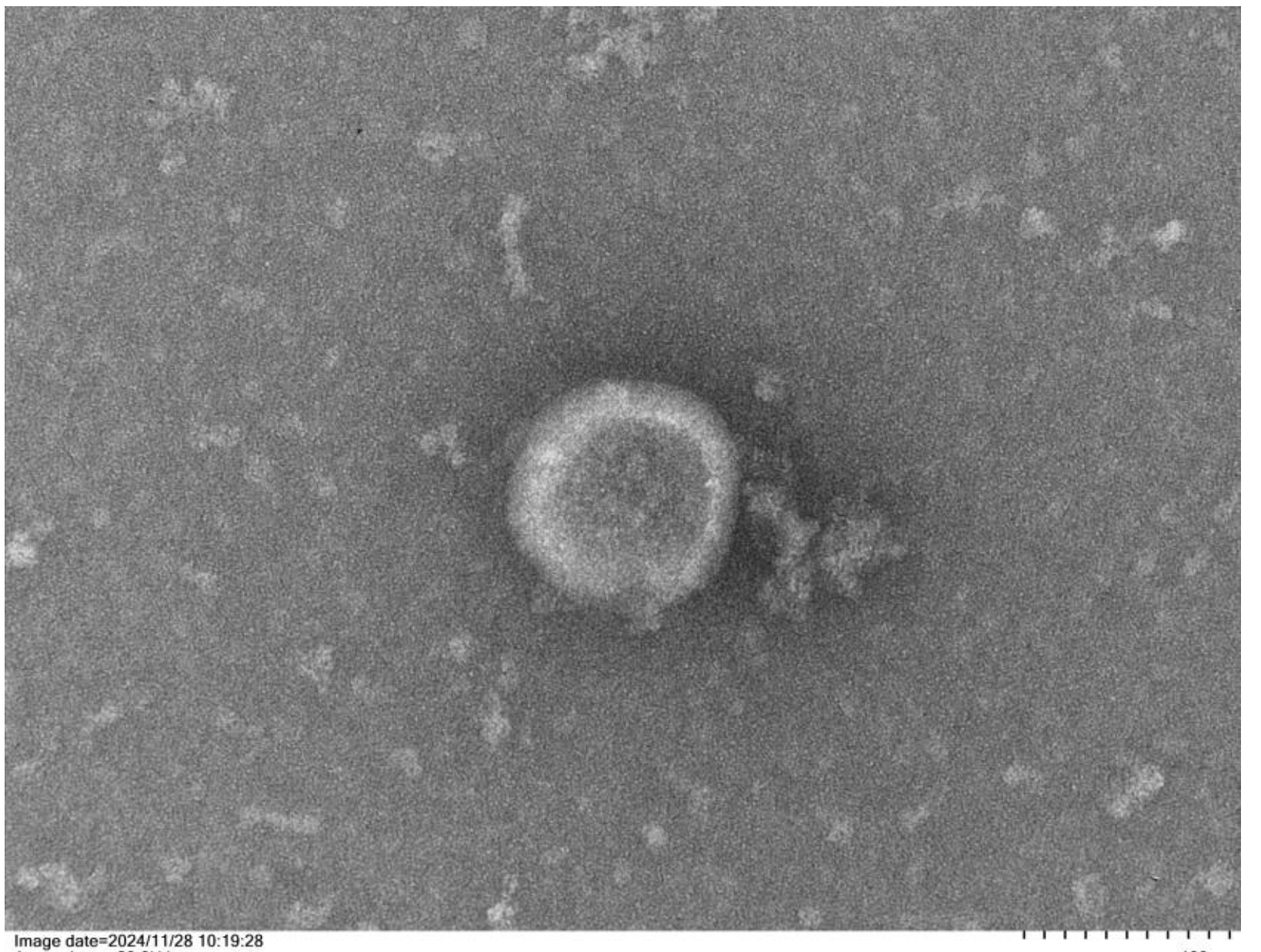

Image date=2024/11/28 10:19:28
Acc. voltage=80.0kV
Magnification=x60.0k

100nm

Original Images for Figure5.A

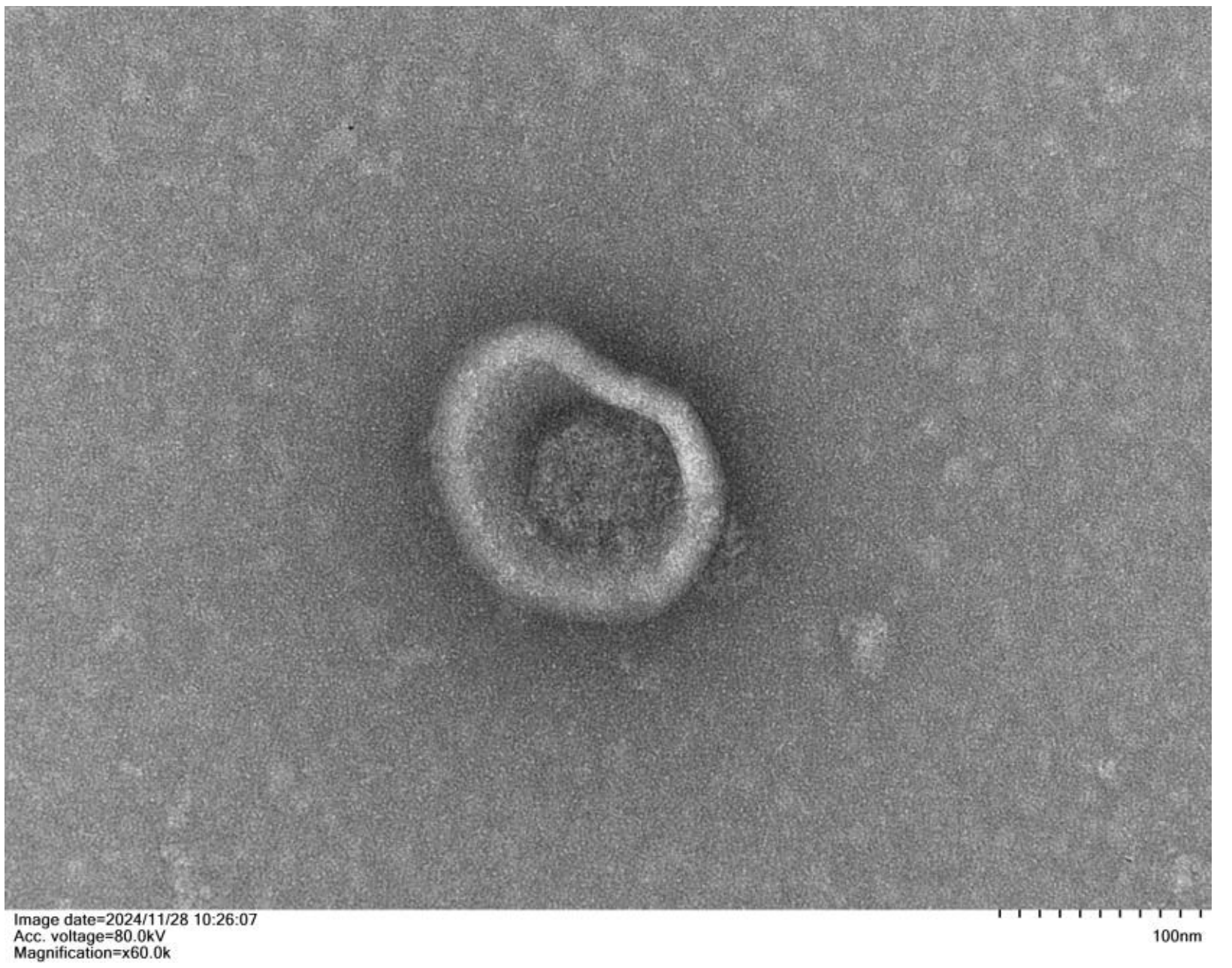

Image date=2024/11/28 10:26:07
Acc. voltage=80.0kV
Magnification=x60.0k

100nm

Original Images for Figure3.C, Figure5.C

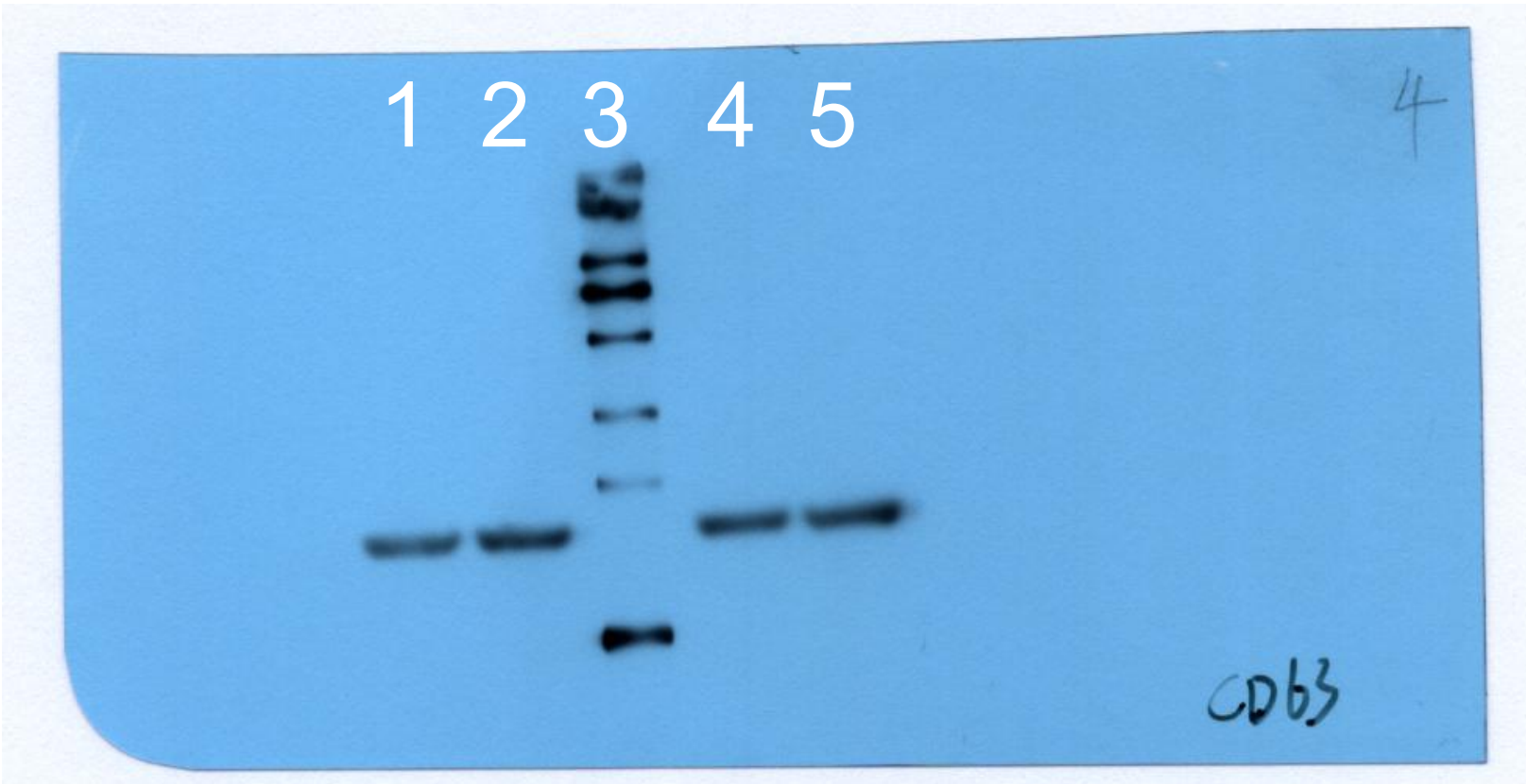

Figure3.C
CD63
1. control;
2. model;
3. marker.

Figure5.C
CD63
3. marker;
4. control;
5. model.

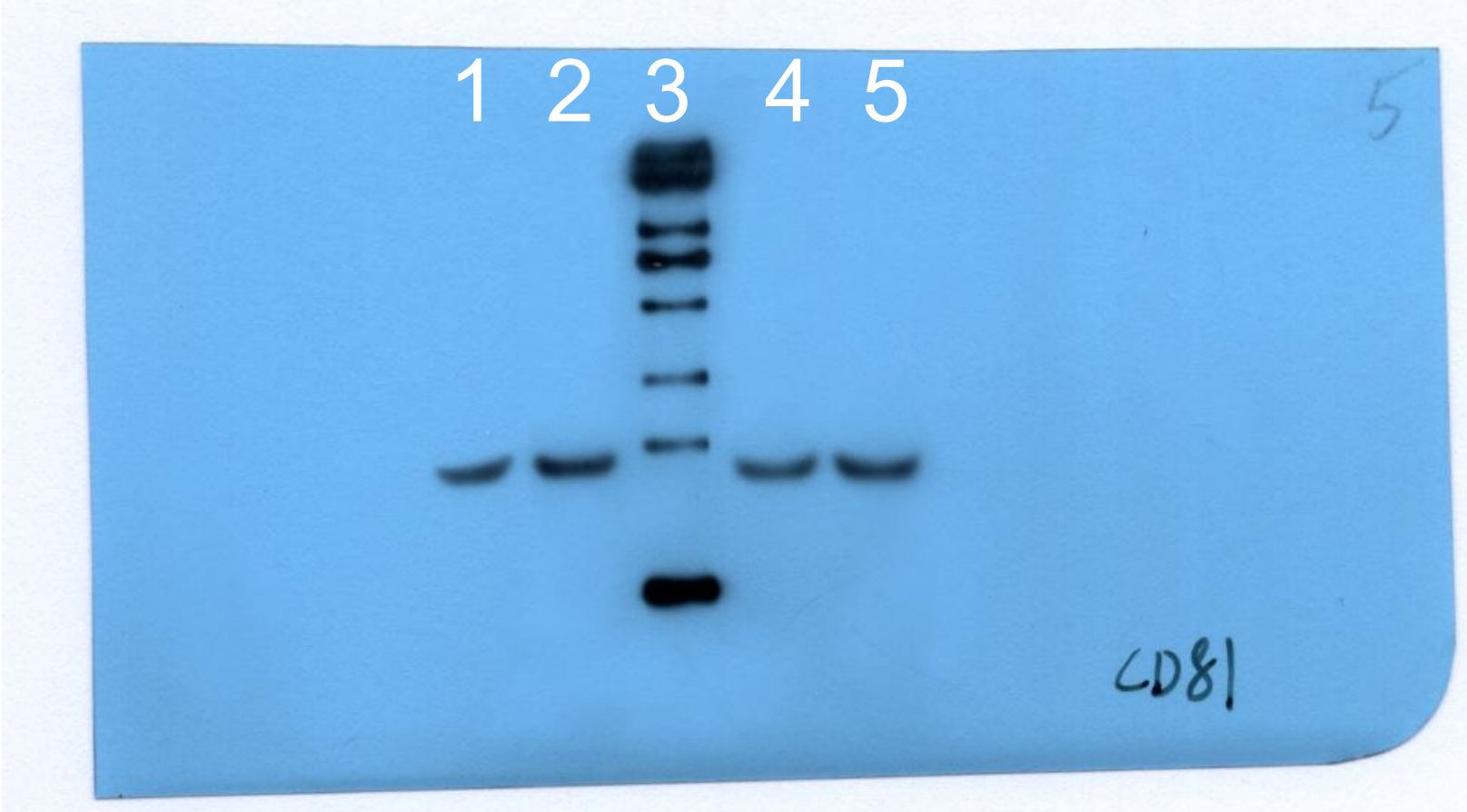

Figure3.C
CD81
1. control;
2. model;
3. marker.

Figure5.C
CD81
3. marker;
4. control;
5. model.

Original Images for Figure3.D, Figure4.C, Figure5.D

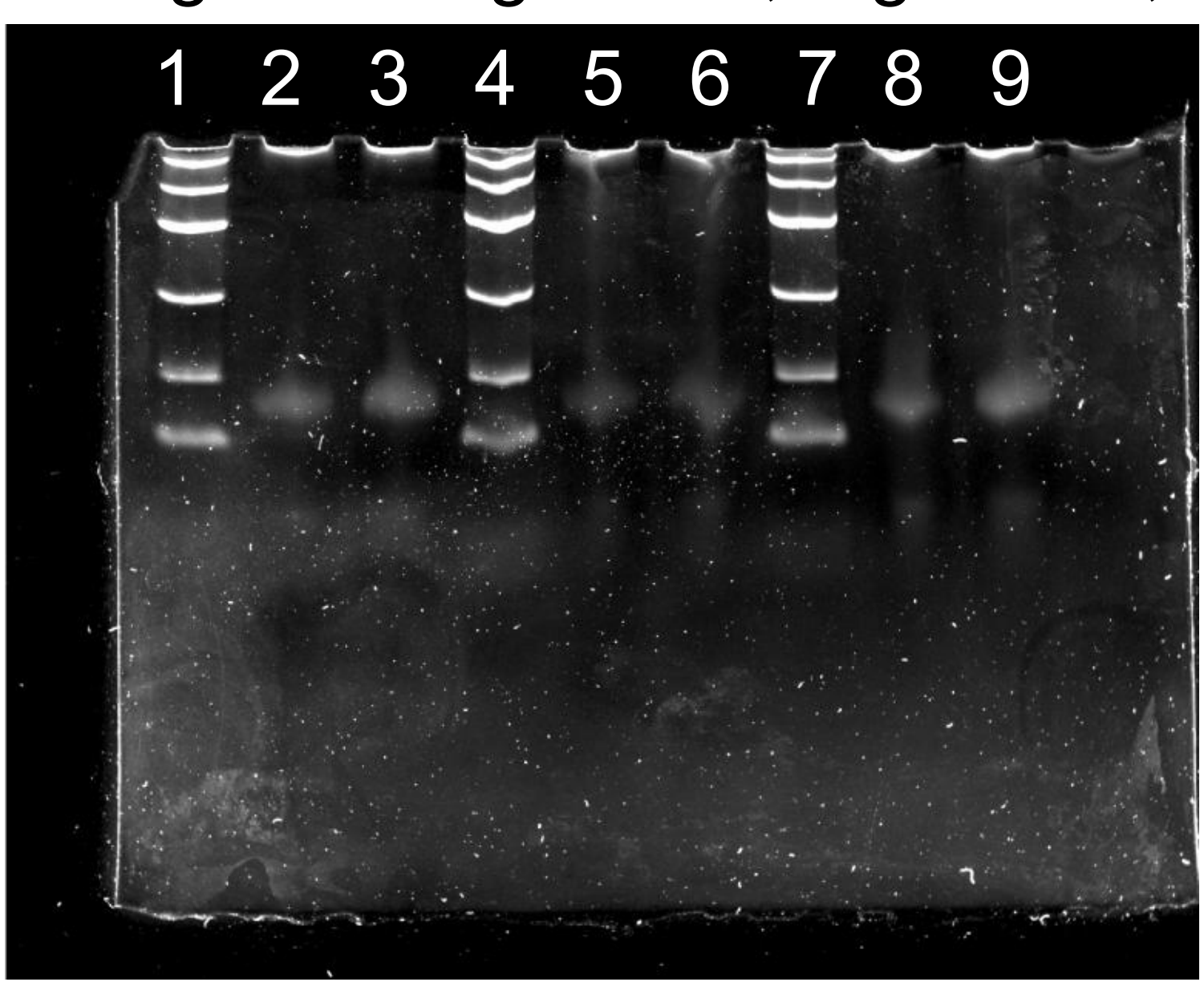

Figure3.D 7. marker; 8. control; 9. model.

Figure4.C 4. marker; 5. control; 6. model.

Figure5.D 1. marker; 2. control; 3. model.
