# Supplementary material for: Abortive transcripts in exosomes - A potential biomarker for Alzheimer’s disease
Source: PLoS One. 2026 Sep 11;21(9):e0357752. doi: 10.1371/journal.pone.0357752 (PMC13567793; doi:10.1371/journal.pone.0357752)
Supplement: Supplementary file 2 [file pone.0357752.s001.zip › Supplementary files/Information on the animals used in this research.pdf]

a.
Animal: Mouse
Strain Name: B6/JGpt-Tg(Thy-APP/Thy-PSEN1)5/Gpt
Strain Abbreviation: FAD4T
Strain Type: Tg
Strain Number: T053302
Strain Background: C57BL/6JGpt
Sex: Male
Age: 33 weeks
b.
The mice used in this study were purchased from GemPharmatech Co., Ltd.
This animal model was developed by transferring the APP gene with human-derived Swedish and Indiana mutations and the human PSEN1 gene with M146V and L286V mutations into the mouse genome.
