# Supplementary material for: Abortive transcripts in exosomes - A potential biomarker for Alzheimer’s disease
Source: PLoS One. 2026 Sep 11;21(9):e0357752. doi: 10.1371/journal.pone.0357752 (PMC13567793; doi:10.1371/journal.pone.0357752)
Supplement: Supplementary file 2 [file pone.0357752.s001.zip › Supplementary files/Supplementary tables.pdf]

Abortive Transcripts In Exosomes - A Potential Biomarker For Alzheimer's Disease

Haizhu Wu, Na Zhang, Weiwei Yan, Cailin Li, Xiaomei Zhang, Xianglin Xv, Fang Fang, Qinshao Wei, Lifeng Zhao

Supplementary Tables

Supplementary Table S1: Antibodies used in western blot

Antibody Name	Manufacturer
HRP-conjugated Monoclonal Mouse Anti-glyceraldehyde-3-phosphate Dehydrogenase (GAPDH)	Aksomics, China
Rabbit monoclonal to CD63 (1:1000)	Abcam, England
Rabbit monoclonal to CD81 (1:10000)	Abcam, England
Goat Anti-Rabbit IgG (H+L) (1:20000)	Southern Biotech, China
Mouse/Human ads-HRP (1:20000)	Southern Biotech, China
Rabbit Anti-Mouse IgG (H+L)-HRP (1:10000)	Southern Biotech, China

Supplementary Table S2: The starting transcription sequence

Gene	Sequence
Gapdh	5′-AACCCUUA-3′
Nefl	5′-ACACAGCC-3′
Bace1	5′-ACAAGCCU-3′
Tyrobp	5′-AGUGCAUA-3′
Ccl2	5′-GCAGAGAG-3′
Pf4	5′-AGAACUUU-3′
Acp2	5′-ACAACAGU-3′
Bcar3	5′-AGUAAGGA-3′
Bnip3	5′-AGAACUGA-3′

Supplementary Table S3: The primer sequences used in RT-qPCR

Gene	Sequence
Gapdh	forward 5′-CATCACTGCCACCCAGAAGACTG-3′
	reverse 5′-ATGCCAGTGAGCTTCCCGTTCAG-3′
Nefl	forward 5′-GCCTTGGACATCGAGATTGCAG-3′
	reverse 5′-CAAGCCACTGTAAGCAGAACGG-3′
Bace1	forward 5′-TGCTGCCATCACTGAATCGGAC-3′
	reverse 5′-GGAATGTGGGTCTGCTTCACCA-3′
Tyrobp	forward 5′-GTGACTTGGTGTTGACTCTGCTG-3′
	reverse 5′-GATAAGGCGACTCAGTCTCAGC-3′
Ccl2	forward 5′-GCTACAAGAGGATCACCAGCAG-3′
	reverse 5′-GTCTGGACCCATTCCTTCTTGG-3′
Pf4	forward 5′-GTTGTTTCTGCCAGCGGTGGTT-3′
	reverse 5′-ACAGTGGCGTCCTGCCTTGATC-3′
Acp2	forward 5′-CTGAACACCTCTTACCACAGGC-3′
	reverse 5′-TCATTGGGAGGGAAGAGTCCAG-3′
Bcar3	forward 5′-ACCACTACACGCAGACAGCCAT-3′
	reverse 5′-AAGGTCACCAGTGGCATCAGCA-3′
Bnip3	forward 5′-GCTCCAAGAGTTCTCACTGTGAC-3′
	reverse 5′-GTTTTTCTCGCCAAAGCTGTGGC-3′

Supplementary Table S4: Adapter RNAs sequence

	Adapter RNAs
upstream	5′-UCACCACUACGCUCACGGCACGAACGCUAUUCCAG-3′
downstream	5′-AUAGGAAGGGAGUGGGCUCACGACCAUGGAUUGGA-3′

Supplementary Table S5: Splint DNAs (SPD) sequence

Gene	SPD
Gapdh	5′-TGAGCCCACTCCCTTCCTATTAAGGGTTCTGGAATAGCGTTCGTGCCG-3′
Nefl	5′-TGAGCCCACTCCCTTCCTATGGCTGTGTCTGGAATAGCGTTCGTGCCG-3′
Bace1	5′-TGAGCCCACTCCCTTCCTATAGGCTTGTCTGGAATAGCGTTCGTGCCG-3′
Tyrobp	5′-TGAGCCCACTCCCTTCCTATTATGCACTCTGGAATAGCGTTCGTGCCG-3′
Ccl2	5′-TGAGCCCACTCCCTTCCTATCTCTCTGCCTGGAATAGCGTTCGTGCCG-3′
Pf4	5′-TGAGCCCACTCCCTTCCTATAAAGTTCTCTGGAATAGCGTTCGTGCCG-3′
Acp2	5′-TGAGCCCACTCCCTTCCTATACTGTTGTCTGGAATAGCGTTCGTGCCG-3′
Bcar3	5′-TGAGCCCACTCCCTTCCTATTCCTTACTCTGGAATAGCGTTCGTGCCG-3′
Bnip3	5′-TGAGCCCACTCCCTTCCTATTCAGTTCTCTGGAATAGCGTTCGTGCCG-3′

Supplementary Table S6: TaqMan-MGB probe sequence

Gene	Sequence
Gapdh	5′-TAAGGGTTCTGGAATAGC-3′
Nefl	5′-CCTATGGCTGTGTCTGG-3′
Bace1	5′-CTATAGGCTTGTCTGGAATAG-3′
Tyrobp	5′-CCTTCCTATTATGCACTCTG-3′
Ccl2	5′-CTATCTCTCTGCCTGGAAT-3′
Pf4	5′-AAAGTTCTCTGGAATAGCGTT-3′
Acp2	5′-CTTCCTATACTGTTGTCTGGAA-3′
Bcar3	5′-CTTCCTATTCCTTACTCTGGA-3′
Bnip3	5′-CTTCCTATTCAGTTCTCTGGA-3′

Supplementary Table S7: The primer sequences used in TaqMan-MGB qPCR

	Primer Sequences
forward	5′-TCACCACTACGCTCACGGC-3′
reverse	5′-TCCAATCCATGGTCGTGAG-3′
